# The Effect of Tempering Temperature on the Microstructure and Properties of a Novel High-Temperature Bearing Steel

**DOI:** 10.3390/ma19020443

**Published:** 2026-01-22

**Authors:** Kai Zheng, Hui Wang, Feng Yu, Shuangping Lin, Zhenqian Zhong, Cunyu Wang, Jianxiong Liang, Wenquan Cao

**Affiliations:** 1 Central Iron and Steel Research Institute (CISRI) of China, Beijing 100081, China; wanghui@nercast.com (H.W.); yufeng@nercast.com (F.Y.); splin1982@cisri.com.cn (S.L.); zhongzhenqian@ncschina.com (Z.Z.); wangcunyu@nercast.com (C.W.);; 2 The NCS Testing Technology Co., Ltd., Beijing 100081, China; 3 State Key Laboratory of Advanced Special Steel, Beijing 100081, China

**Keywords:** M_6_C carbides, M_2_C carbides, reverted austenite, dislocation density, strengthening mechanism

## Abstract

The microstructure, precipitation behavior, and mechanical properties of an ultrahigh-strength stainless bearing steel after tempering were investigated using multiscale characterization techniques along with tensile and impact testing. Based on the experimental results, strengthening and toughening mechanisms are discussed. The findings indicate that in samples tempered between 450 °C and 540 °C, tensile strength increases while impact toughness decreases. This is primarily attributed to the precipitation of M_6_C and M_2_C carbides and a reduction in dislocation density. In contrast, after tempering at 580 °C, the formation of increasing amounts of thick film-like reverted austenite along lath and twin boundaries results in a slight decline in tensile strength accompanied by improved elongation. The dominant strengthening mechanism for samples tempered between 450 °C and 500 °C is the synergistic effect of dislocation strengthening and precipitation strengthening. Above 520 °C, precipitation strengthening becomes the primary mechanism. However, the coarsening of acicular or lamellar M_2_C carbides during precipitation appears to significantly degrade toughness.

## 1. Introduction

The continuous advancement of the aviation industry has led to increasingly demanding operating conditions for engine components, involving higher loads, elevated temperatures, and increased rotational speeds [1]. Correspondingly, next-generation aircraft engines demand bearing steels that combine high temperature resistance, corrosion resistance, and high surface hardness, with a core that retains good ductility and toughness [2,3]. At present, the most commonly employed bearing steels are high-carbon bearing steels and carburized bearing steels [4]. The representative and currently widely used high-carbon bearing steel, such as M50, BG42, Cronidur 30 [5,6,7], has good wear resistance and high strength, yet it is deficient in fracture toughness. M50NiL and Pyrowear 675 as carburized bearing steels are developed for excellent fracture toughness in the core, but they are still inadequate in wear resistance and corrosion resistance [7,8,9,10]. The recently developed ultrahigh-strength stainless bearing steel, as represented by CSS-42L steel, is alloyed with low C but high Cr, Co, and Mo to improve corrosion resistance, while guaranteeing good fracture toughness [11,12,13,14].

The typical chemical composition of conventional CSS-42L stainless steel is Fe-13.84Cr-12.48Co-4.67Mo-2.11Ni-0.60V-0.16Mn-0.15Si-0.13C-0.04Nb (wt.%) [14,15]. In comparison, a novel alloy has been developed by increasing the contents of V and C and introducing the element W. The novel alloy has a chemical composition of Fe–14Cr–13Co–5Mo–2Ni–1W–1V–0.16C–0.12Nb–0.074Si–0.027Mn (wt.%) [16,17]. After case-carburizing, it exhibits an ultrahigh surface hardness of 71 HRC and an excellent through-depth hardness gradient. It also demonstrates superior surface hardness within the 200–400 °C temperature range when compared to other advanced high-temperature bearing steels, including M50, M50NiL, and conventional CSS-42L steels. These alloying composition modifications have demonstrably conferred beneficial effects on both the microstructure and performance of the carburized layer. For the successful engineering application of this novel steel, investigating the core microstructure evolution and strengthening–toughening mechanisms is equally critical. Chen et al. [2] and Chen et al. [11] found that M_6_C and M_2_C carbides were the main precipitates during 490–550 °C tempering and 530 °C long-term tempering in CSS-42L steel. Xiao et al. [18] observed a large number of stable spherical M_6_C carbides precipitated in a Cr15Co10Mo5 steel (modified variant of CSS-42L bearing steel), tempered at 540 °C, and M_6_C, M_2_C and M_23_C_6_ carbides in the sample tempered at 600 °C. Whether the microstructural evolution and strengthening–toughening mechanisms of this novel steel during tempering align with those of conventional CSS-42L remains unclear and requires systematic investigation.

Previous studies have investigated the effects of quenching temperature on the core microstructure and mechanical properties of this steel [17,19]. This study further investigates the influence of tempering temperature on both the mechanical properties and microstructure of the novel steel. On this basis, the influence of tempering temperature on the strength and toughness mechanism is analyzed and discussed. This study aims to provide theoretical foundations for the engineering application of this novel high-temperature bearing steel, while offering valuable references for alloy design optimization of similar carburized high-temperature bearing steels.

## 2. Materials and Methods

**Figure 1:**
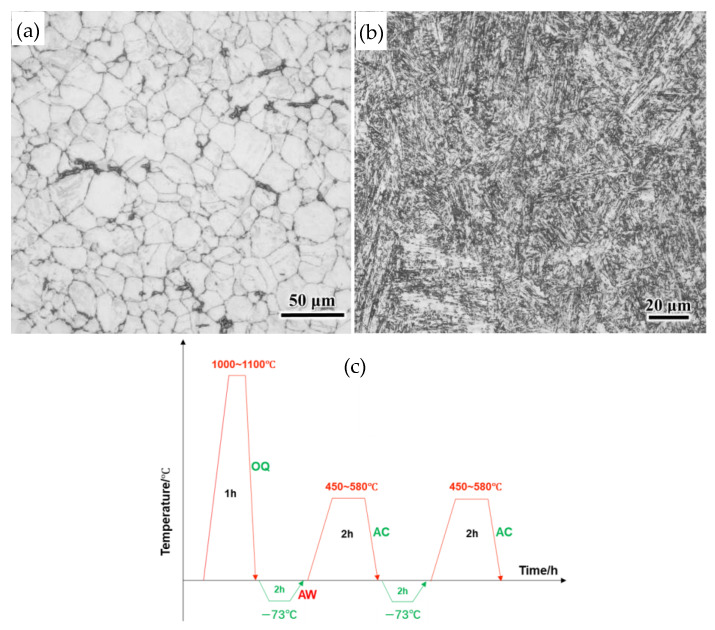
Optical microscope (Oberkochen, BW, Germany) microstructure images before (**a**) and after heat treatment (**b**); heat treatment process flowchart (**c**) [19].

The experimental steel had a nominal chemical composition (wt.%) of C 0.17, Co 13.0, Cr 14.0, Mo 5.0, Ni 2.0, W 1.0, V 1.0, Nb 0.1, Si 0.1, Mn 0.03, and Fe balance. It was produced through vacuum induction melting and vacuum arc remelting; this was followed by forging it into 90 mm diameter bars and furnace cooling it to room temperature. The resulting microstructure consists of fine ferrite with carbides distributed along grain boundaries, as shown in Figure 1a. Samples were sectioned longitudinally from the forged bar, austenitized at 1050 °C for 1 h, and then oil quenched. Subsequently, they were held at−73 °C for 2 h, air heated to room temperature, and tempered between 450 and 580 °C for 2 h. The cryogenic treatment and tempering cycle were repeated once. The heat-treated samples were designated as T450 to T580, where the numerical suffix indicates the tempering temperature. The metallographic structure of the sample after tempering (T500) exhibits uniform and fine-tempered martensite, as shown in Figure 1b. A flowchart of the heat treatment process is provided in Figure 1c.

Phase volume fractions and dislocation densities of all tempered samples were measured using a Bruker D8 ADVANCE X-ray diffractometer (Billerica, MA, USA) with a Co target. The scanning parameters were set as follows: a range of 45–115°, a tube current of 40 mA, a tube voltage of 35 kV, and a scan speed of 2°/min. Details of the methods used to calculate the austenite volume fraction and dislocation density, which follow established procedures described in references [20,21], have been previously reported in reference [19].

For microstructure characterization, a metallographic etchant with the following composition was used: 1 g CuCl_2_, 3.5 g FeCl_3_, 2.5 mL HNO_3_, 50 mL C_2_H_5_OH, 50 mL HCl, and 50 mL H_2_O. The microstructural morphology of T500 was examined using a JEOL JSM-IT800 (Tokyo, Japan) scanning electron microscope (SEM). Electron backscatter diffraction (EBSD) analysis was performed on electrochemically polished samples of T520, T550, and T580, with a step size of 100 nm and an acceleration voltage of 20 kV. The collected EBSD data were processed using the commercial HKL Channel 5 software. For transmission electron microscopy (TEM) observations, the samples were ground to approximately 50 μm in thickness and subsequently thinned using a TJ100-SE twin-jet electrophisher (Shenyang, China). The electrolyte consisted of 6% HClO_4_ in ethanol and was maintained at a temperature between −25 °C and −30 °C. The microstructure and precipitates were examined using an FEI Tecnai G2 F20 (New York, NY, USA) transmission electron microscope (TEM), equipped with an Oxford Instruments (Abingdon, Oxon, UK) energy-dispersive X-ray spectroscopy (EDS) detector and operated at an accelerating voltage of 200 kV.

Dog-bone-shaped tensile specimens with a gauge dimension of Φ5 mm × 30 mm were prepared for tensile testing. Tensile tests were performed on a Zwick Z250rTL testing machine (Ulm, Bavaria, Germany) in accordance with the Chinese national standard GB/T 228.1-2021 [22] to assess strength and ductility. Impact tests were conducted using U-notch Charpy specimens (10 mm × 10 mm × 55 mm, notch depth = 2 mm) on a Wance PIT752H impact tester (Shenzhen, China). To ensure statistical reliability, three parallel specimens were tested for both tensile and Charpy impact tests. Rockwell hardness was measured using a Wilson RB2000-T hardness tester (Norwood, MA, USA), with the average value for each sample derived from five valid measurements.

## 3. Results

### 3.1. Microstructure Characterization

**Figure 2:**
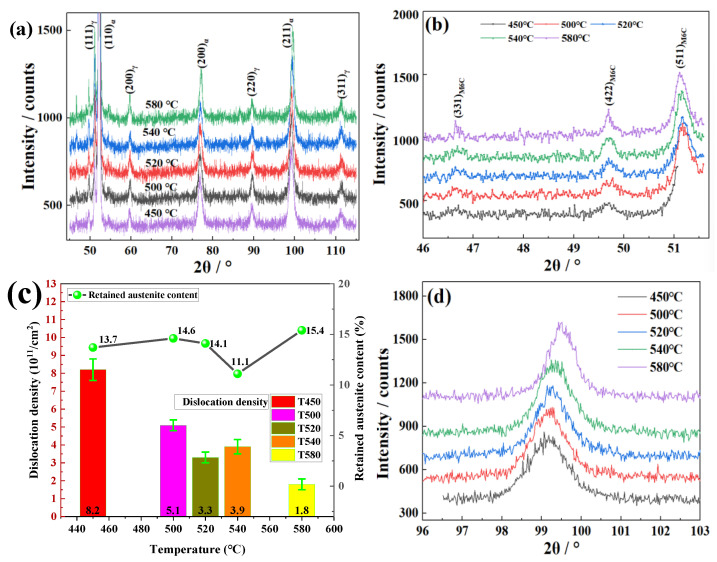
XRD diffraction patterns and analysis: (**a**) martensite and austenite crystal plane calibration; (**b**) M_6_C carbide crystal plane calibration; (**c**) changes in retained austenite content and dislocation density with tempering temperature; (**d**) (211) crystal plane diffraction peak of martensite.

Figure 2a shows XRD diffraction patterns. It can be seen from Figure 2a,b that the phases of T450-T580 consist of martensite, austenite, and a bit of M_6_C carbides. The diffraction peaks corresponding to the martensite and austenite crystal planes are calibrated in Figure 2a and that of M_6_C carbides are calibrated in Figure 2b.

The austenite volume fraction of samples treated at 450 °C, 500 °C, 520 °C, 540 °C, 580 °C are calculated as 13.7%, 14.6%, 14.1%, 11.1% and 15.4%, respectively. The plot of the austenite content with tempering temperature is shown in Figure 2c. When the tempering temperature is between 450 °C and 520 °C, the austenite content remains almost unchanged around 14% as the tempering temperature increases. When the tempering temperature continues to rise to 540 °C, the austenite content rapidly decreases to 11.1%, but rapidly rises to 15.4% with the temperature rising to 580 °C.

The dislocation density of samples treated at 450 °C, 500 °C, 520 °C, 540 °C, 580 °C are calculated as 8.2 × 10^15^/m^2^, 5.1 × 10^15^/m^2^, 3.3 × 10^15^/m^2^, 3.9 × 10^15^/m^2^, 1.8 × 10^15^/m^2^, respectively. The plot of the dislocation density with tempering temperature is also shown in Figure 2c. Except for a slight increase in dislocation density during tempering at 540 °C, the dislocation density gradually decreases as the tempering temperature increases. It can be seen from Figure 2d that the FWHM of the (211) diffraction peaks decreases with increasing tempering temperature.

**Figure 3:**
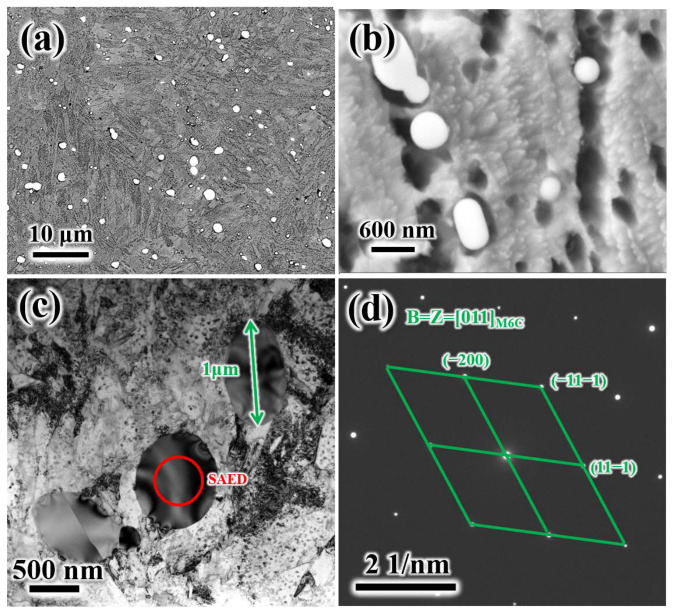
Microstructure morphology: (**a**,**b**) SEM morphology granular carbides in T500; (**c**,**d**) TEM morphology and SAED pattern of undissolved granular secondary carbides.

SEM microstructure morphology of T500 shows the M_6_C carbides presenting a granular shape. Based on this observation, it is inferred that these granular carbides are predominantly undissolved secondary carbides that persisted through the austenitization process. The undissolved secondary carbides (about 1 μm) were also determined to be M_6_C carbides on the basis of selected area electron diffraction (SAED) (Figure 3c,d).

**Figure 4:**
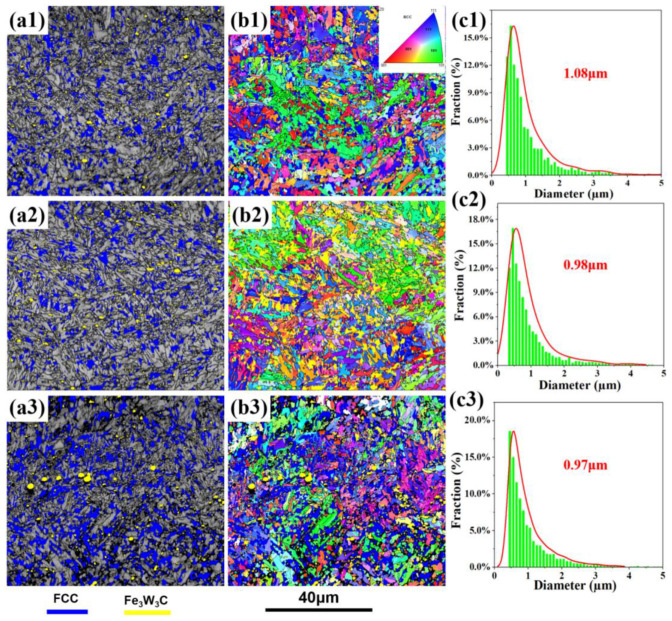
EBSD analysis results of T520 (**a1**–**c1**), T540 (**a2**–**c2**), T580 (**a3**–**c3**): (**a1**–**a3**) phase distribution map of electron backscatter diffraction (EBSD), with the M_6_C marked with yellow and austenite marked with blue; (**b1**–**b3**) inverse pole figure (IPF); coloring orientation images; (**c1**–**c3**) the grain size distribution.

Figure 4 and Table 1 show EBSD analysis results of T520, T540 and T580, respectively. The content of granular M_6_C remains almost unchanged with increasing tempering temperature, and the size of blocky shape austenite coarsens obviously as tempering temperature increases from 540 °C to 580 °C. Except for that, changes in martensite substructure and crystallographic orientation are observed. Inverse pole figure (IPF) orientation maps of the tempered microstructures are shown in Figure 4(b1–b3). As the tempering temperature increases from 520 °C to 580 °C, the average block width remains largely unchanged. During EBSD data processing, grain size was determined using a grain tolerance angle of 15°. Table 2 lists the calculated average block diameters for the steel tempered at 520 °C, 540 °C, and 580 °C. The corresponding grain size frequency distributions are presented in Figure 4(c1–c3). The results indicate that the peak around 0.6 μm and the average diameter both remain at approximately 1.0 μm.

**Figure 5:**
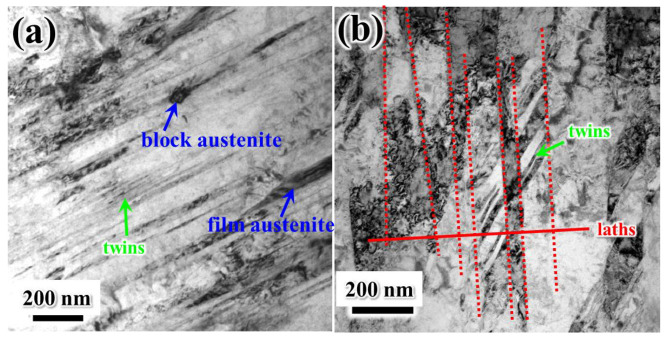
TEM images of austenite in T500: (**a**) morphology of block austenite, film austenite, and twins; (**b**) lathes and twins. The position marked by the red dashed line is the martensite lathes boundaries.

Figure 5 presents TEM images of the T500 sample. A significant amount of twinning is observed within the martensitic matrix, which is characteristic of secondary hardening steels with a low martensite start temperature (M_s_) [23,24]. The average width of the martensite lamellae in T500 was measured to be approximately 107 nm (Figure 5b). Additionally, two distinct types of austenite with differing morphologies were identified: blocky austenite and filmy austenite. The blocky austenite, commonly referred to as retained austenite, results from incomplete transformation during the quenching process. In contrast, the filmy austenite is considered to be reverted austenite, formed during tempering via a recrystallization mechanism [25].

**Figure 6:**
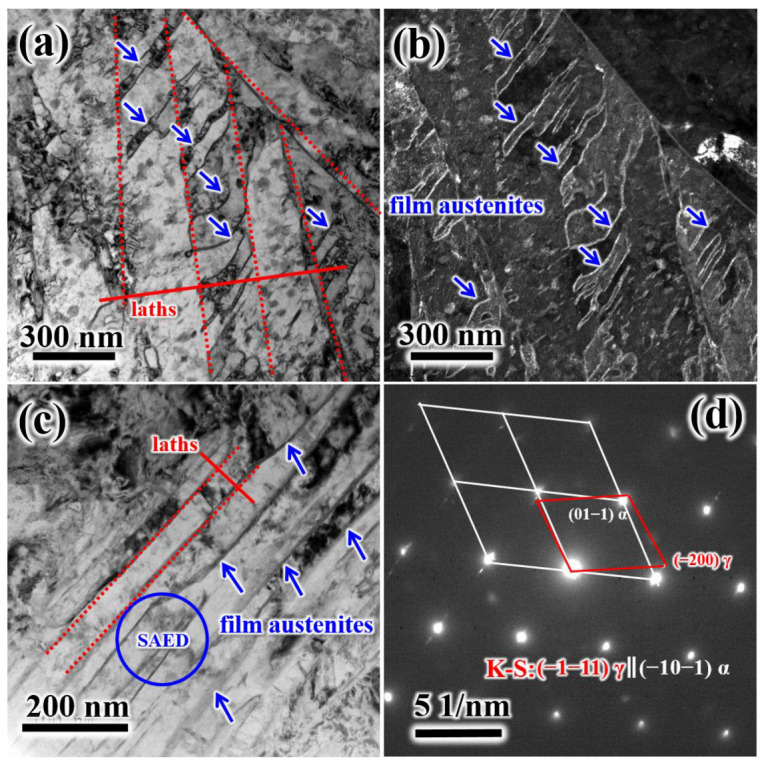
TEM images of T580: bright field (**a**) and dark field. (**b**) TEM images of martensite lamellae and film austenite at twin boundaries; (**c**) bright field TEM images of martensite lamellae and film austenite at laths boundaries; (**d**) SAED pattern and indexing of the martensite and film austenite (blue circle, Figure 6c). The position indicated by the blue arrow is the film-like reversed austenite.

Figure 6 presents TEM images of T580. The average width of the martensite lamellae in T580 was measured to be approximately 200 nm (Figure 6a). As the tempering temperature increases, the blocky retained austenite gradually decomposes, while film-like reverted austenite forms [2,11,26]. At a tempering temperature of 580 °C, increasingly thicker film austenite develops along lath boundaries and twin boundaries, as illustrated in Figure 6a–c. The corresponding selected-area electron diffraction (SAED) pattern of the reverted austenite in T580 is shown in Figure 6d. The film-reverted austenite maintains a Kurdjumov–Sachs (K–S) orientation relationship with the martensite matrix, specifically (−1−11)_γ_ ‖ (−10−1)_α_ and [011]_γ_ ‖ [111]_α_, which is consistent with previously reported results [25].

**Figure 7:**
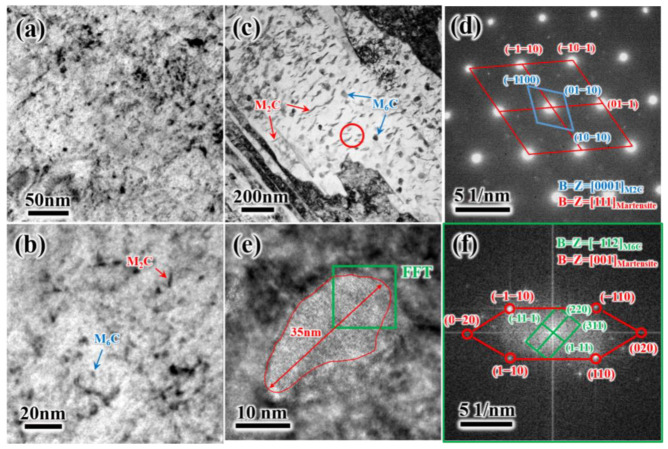
TEM images showing needle- or strip-granular-shaped precipitates in T520 and T580: (**a**,**b**) morphology of precipitates in T520; (**d**) SAED pattern of needle- or strip-shaped precipitates M_2_C indicated by red circle in (**c**); (**e**) HRTEM image of granular shape precipitates M_6_C; (**f**) calibration of granular shape precipitates M_6_C and martensite matrix in (**e**). The red line in Figure (**e**) indicates the interface between the precipitates M_6_C and the martensite matrix.

Figure 7 shows precipitates in T500 and T580. As seen in Figure 7a,b that there are two types of precipitates, namely needle- or strip-like precipitates and granular spherical shape precipitates. With tempering temperature increasing to 580 °C, precipitates grow significantly. Selected-area electron diffraction (SAED) analysis on needle or strip precipitates marked by the red circle in Figure 7c proved that this type of carbide is M_2_C carbide. The granular spherical shape precipitates with lengths of about 35 nm are determined to be M_6_C carbides on the basis of high-resolution transmission electron microscopy (HRTEM) analysis (Figure 7e,f).

### 3.2. Mechanical Properties

**Figure 8:**
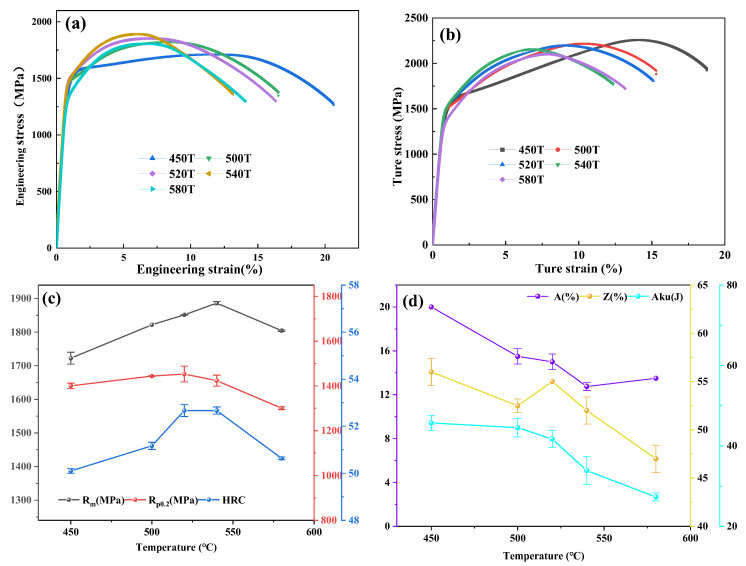
The engineering stress–strain curves (**a**) and true stress–strain curves (**b**) of samples tempered at different temperature; plots of hardness, tensile strength, yield strength, elongation, reduction area, and impact absorbed energy versus tempering temperature (**c**,**d**).

Figure 8a,b presents the engineering and corresponding true stress–strain curves for the tested steels. As illustrated in Figure 8a, the ultimate tensile strength increases while the uniform elongation decreases with rising tempering temperature. Figure 8b displays the true stress–strain curves, from which it can be observed that the true ultimate tensile strengths of the T450–T580 samples are 2258 MPa, 2216 MPa, 2193 MPa, 2158 MPa, and 2104 MPa, with corresponding total true tensile strains of 18.8%, 15.3%, 15.1%, 12.4%, and 13.2%, respectively. Both the true ultimate tensile strength and total true strain generally decrease with increasing tempering temperature, except for the sample tempered at 580 °C, which exhibits a slightly higher total true strain compared to the sample treated at 540 °C.

Table 2 summarizes the mechanical properties of the T450-T580 samples. And the variation curve with tempering temperature is shown in Figure 8c,d. For tempering temperatures lower than 540 °C, the tensile strength (TS, Rm) increases nearly linearly from 1723.0 MPa to 1887.0 MPa, while the yield strength (YS, Rp_0_._2_) fluctuates within the range of 1400–1450MPa. As the tempering temperature increases to 580 °C, the tensile strength (TS) declines sharply to 1805.0 MPa, and the yield strength (YS) decreases substantially to 1301 MPa. The Rockwell hardness (HRC) exhibits a trend similar to that of the TS with rising tempering temperature, except that it remains constant at 52.7 HRC within the 520–540 °C range. Over the entire tempering temperature range studied, the section shrinkage (Z) shows an almost linear decrease from 56.0% to 47.0% with increasing temperature, except for the sample tempered at 520 °C, which displays higher section shrinkage than those treated at 500 °C and 540 °C. In contrast, the elongation (A) follows an opposite trend to the tensile strength: it decreases from 20.0% to 12.8% at tempering temperatures below 540 °C, then rises to 13.5% when the temperature reaches 580 °C.

The impact absorbed energy (**A_ku_**) decreases with tempering temperature, over the temperature range of 450 °C to 520 °C, as the **A_ku_** values slowly decrease from 45.7 J to 41.7 J. As the tempering temperature is raised to 540 °C and 580 °C, the **A_ku_** values decrease significantly to 33.9 J and 27.3 J, respectively.

## 4. Discussion

### 4.1. Microstructure Evolution

**Figure 9:**
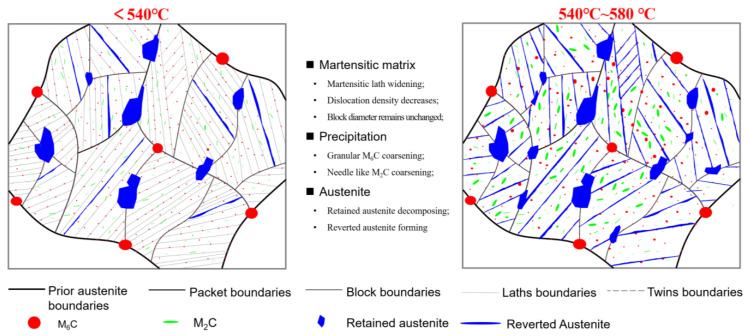
Schematic diagram for microstructure evolution during tempering at different temperatures.

As a summary, the schematic diagram for microstructure evolution during tempering at different temperatures is shown in Figure 9. As the tempering temperature increases, the diffusion rate of C increases and the supersaturated martensite accelerates the precipitation of carbides which are calibrated as M_2_C and M_6_C in Figure 7. An increase in tempering temperature will accelerate the climb and submergence of dislocations, resulting in a decreasing trend in dislocation density. An increase in the number of precipitates will enhance the pinning effect on dislocations, but as the precipitates coarsen, they will weaken the pinning effect on dislocations. The slight increase in dislocation density during tempering at 540 °C may be closely related to the number and size of precipitates.

**Figure 10:**
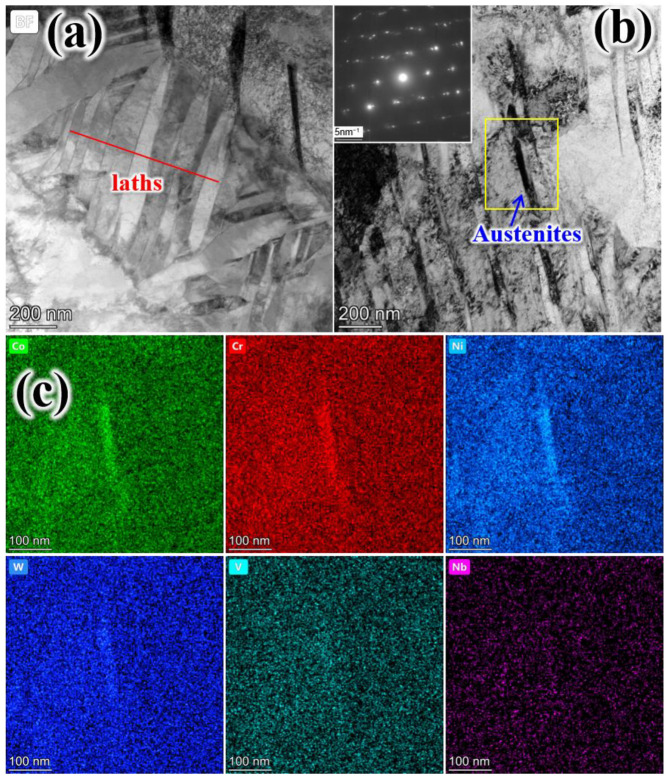
Morphology (**a**,**b**) and elemental mapping (**c**) of martensite laths and retained austenite in T520.

The change in austenite during tempering is mainly affected by the decomposition of the retained austenite and the formation of reversed austenite [26]. The decomposition of the retained austenite becomes faster with increasing temperature. The reason is that, on the one hand, the conditioning of retained austenite becomes faster. On the other hand, austenite instability is caused by the carbon concentration reduction due to carbide precipitation [11]. Figure 10 shows the morphology and elemental mapping of the martensite laths and austenite in the T520 sample, revealing that elements such as Cr, Co, Ni, W, and V segregate to the austenite. Micro-segregation of austenite-stabilizing elements (e.g., Cr, Co, Ni) within the martensite shifts the local phase equilibrium. This allows austenite to form in these enriched zones despite the bulk tempering temperature being below A_c1_ [25]. Additionally, a higher tempering temperature provides a greater driving force for reversed austenite formation, thereby yielding a higher volume fraction.

### 4.2. Strengthening and Toughening Mechanism

The strength of martensitic ultrahigh-strength steel can be approximately rationalized as the cumulative contribution of various strengthening mechanisms, including solid solution strengthening, grain boundary strengthening, dislocation strengthening, and precipitation strengthening [20,27]. The following equations are applied for quantification (all formulas and calculations adhere strictly to the International System of Units (SI) for standardization):

*σ* = *σ_0_* + *σ_ss_* + *σ_d_* + *σ_p_* + *σ_g_* (1)

Here, *σ* represents the total strength, with *σ*_0_ denoting the internal frictional stress of body-centered cubic (BCC) iron, approximately 50 MPa [27]. *σ_d_* arises from high dislocation density (dislocation strengthening), *σ_g_* originates from grain boundaries (fine-grain strengthening), *σ_ss_* results from solid solutes (solid-solution strengthening), and *σ_p_* is contributed by precipitates (precipitation strengthening).

The solid-solution strengthening contribution *σ_ss_* is relatively low, reported as 92 MPa in Ref. [27]. For fine-grain strengthening, the strength increment *σ_g_* is described by the Hall–Petch relationship, *σ_g_* = *k_HP_·d*^−1/2^, where *k_HP_* is the Hall–Petch slope (120 MPa·µm^1/2^) [19,27] and d is the width of the martensite lamellae. Based on this relation, the *σ_g_* values for the T500 and T580 samples are calculated to be 366.9 MPa and 268.3 MPa, respectively.

Nanoscale precipitation and dislocation strengthening appear to contribute more significantly to the overall strength [26,27]. The dislocation strengthening component *σ_d_* can be estimated using the Taylor hardening relationship [20,27]:

*σ_d_* = *MαGb*√*ρ*, (2)

where *M* is the Taylor factor (taken as 2.8 for BCC metals), α is a constant equal to 0.20 for materials with high dislocation density, *G* represents the shear modulus (80.7 GPa), *b* denotes the magnitude of the Burgers vector (0.248 nm), and ρ stands for the dislocation density.

**Figure 11:**
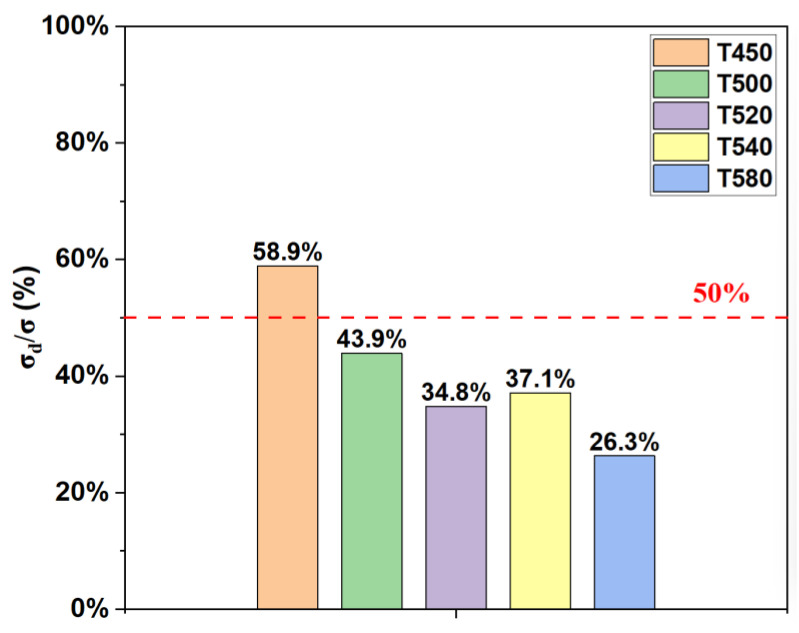
Dislocation strengthening contribution fractions *σ_d_/σ* of samples tempered at different temperatures.

According to Equation (2), *σ_d_* of T450, T500, T520, T540, and T580 are calculated to be 1014.9 MPa, 800.3 MPa, 643.8MPa, 699.9 MPa, and 475.5 MPa, respectively. And the corresponding dislocation strengthening contribution fractions *σ_d_*/*σ* are calculated as 58.9%, 43.9%, 34.8%, 37.1%, and 26.3%, as shown in Figure 11. It can be seen that dislocation strengthening gradually weakens as the tempering temperature increases.

Precipitation hardening arises from the interaction force between individual precipitate particles and dislocations [2]. The capacity of precipitate particles cutting to Orowan looping is determined by the hardness and the spatial distribution of the precipitated particles, as described by the Orowan–Ashby equation:

(3) $$∆\tau_{orowan}=(\frac{Gb}{r})f^{\frac{1}{2}}\ln\left(\frac{r}{b}\right)$$

where *r* denotes the average particle radius, *f* represents the volume fraction of particles, *G* is the shear modulus, and *b* is the magnitude of the Burgers vector.

Okayasu et al. [28] noted that the XRD peak position is closely correlated with the interatomic spacing in the BCC structure. As the tempering temperature increases, the (211) diffraction peak shifts toward higher 2θ values (Figure 2d), indicating a reduction in lattice spacing. This suggests that solute atoms are released from the octahedral interstices of the BCC Fe lattice and subsequently combine with alloying elements to form carbides [11]. Therefore, the volume fraction of precipitates (f) increases with the tempering temperature. Although the average radius of particles also increases simultaneously, which is not good to strengthen, the total precipitation strengthening effects seem to strengthen at the temperature range of 490–550 °C [2]. This is also proven by the increase in tensile strength because the dislocation and grain boundary strengthening become weaker with tempering temperature increasing.

Based on the above analysis, the main strengthening mechanism at tempering temperatures of 450–500 °C is the comprehensive strengthening of dislocation strengthening and precipitation strengthening, and the main strengthening mechanism at tempering temperatures of 520–580 °C is precipitation strengthening.

As shown in Figure 2d, the narrowing of diffraction peaks and their shift toward higher angles confirm both the reduction in lattice distortion and the annihilation of dislocations, which are expected to be enhanced at higher tempering temperatures. These changes facilitate dislocation slip and are generally beneficial to toughness. However, the observed decrease in elongation and impact absorbed energy suggest that martensite recovery is not the predominant factor controlling toughness in these samples. Instead, greater emphasis should be placed on the influence of carbides and austenite on the toughness of the experimental steel.

Austenite exhibits superior plasticity primarily due to its face-centered cubic structure, which provides a greater number of slip systems. When retained at martensite lath boundaries, austenite can act to either trap or inhibit the propagation of cracks [29]. But compared with elongation and impact absorbed energy (Figure 8d), the change in austenite content (Figure 2c) is not significant as tempering temperature increases, especially at 450–520 °C, while carbides grow significantly and coarsen as the tempering temperature increases. Zhang et al. [30] found that after tempering 42CrMo4 high-strength steel at 500–650 °C, the carbides transitioned from an uneven lamellar distribution to a dispersed granular distribution with increasing tempering temperature, resulting in a significant improvement in impact toughness. In this test, steel granular M_6_C carbides exhibited minimal impact on impact toughness, while acicular or lamellar M_2_C carbides served as stress concentration sites that readily initiated crack formation, consequently deteriorating toughness.

## 5. Conclusions

In this study, the microstructure and properties of a novel high-temperature bearing steel subjected to different tempering temperatures are investigated. The strengthening and toughening mechanisms are discussed. The main conclusions can be drawn as follows:

1. As the tempering temperature increases, both needle-shaped M_2_C and granular M_6_C carbides precipitate from the supersaturated martensite and subsequently undergo coarsening, while maintaining coherency with the matrix. Concurrently, the austenite content fluctuates between approximately 13% and 15%, which is attributed to the decomposition of blocky retained austenite and the formation of film-like reversed austenite at lath and twin boundaries. The nucleation and growth of reversed austenite during tempering below Ac_1_ are attributed to the segregation of austenite-stabilizing elements (Cr, Co, Ni). Meanwhile, the dislocation density exhibits a gradual decline.
2. As the tempering temperature increases from 450 °C to 580 °C, the material exhibits a significant trade-off relationship between strength and ductility. In the low-temperature tempering stage (≤540 °C), the tensile strength increases nearly linearly with rising temperature, while elongation and reduction in area continuously decrease. When the tempering temperature reaches 580 °C, the strength indicators (tensile strength, yield strength) significantly decrease, while the elongation shows a slight recovery. The impact toughness decreases in a stepwise manner with increasing tempering temperature, particularly exhibiting a sharp deterioration in the temperature range above 540 °C.
3. The variations in dislocation density, precipitate evolution, and austenite content are the primary factors governing strength and toughness. At 450–500 °C, the dominant strengthening mechanism is a synergistic effect of dislocation strengthening and precipitation hardening. The main strengthening mechanism at tempering temperatures of 520–580 °C is precipitation strengthening. The coarsening of acicular or lamellar M_2_C carbides during precipitating seems to have great negative effects on toughness.

## Figures and Tables

**Table 1:** Block diameter and volume fraction of austenite and M_6_C carbides in the experimental steel tempered at various temperatures.

Temperature (°C)	Block Diameter (μm)	Volume Fraction of M_6_C (%)	Volume Fraction of Austenite (%)
520 °C	1.08 ± 0.05	0.98 ± 0.08	12.3 ± 0.5
540 °C	0.98 ± 0.02	1.40 ± 0.10	14.4 ± 0.8
580 °C	0.97 ± 0.03	1.50 ± 0.15	20.2 ± 1.0

**Table 2:** Mechanical properties of samples T450–T580.

Sample	TS (R_m_)/MPa	YS(R_p0.2_)/MPa	A/%	Z/%	A_ku_/J
T450	1723.0 ± 13.0	1401.0 ± 9.0	20.0 ± 0.0	56.0 ± 1.0	45.7 ± 1.3
T500	1822.0 ± 0.0	1444.0 ± 1.0	15.5 ± 0.5	52.5 ± 0.5	44.6 ± 1.7
T520	1851.0 ± 0.5	1453.0 ± 25.0	15.0 ± 0.5	55.0 ± 0.0	41.7 ± 1.5
T540	1887.0 ± 3.0	1424.0 ± 18.0	12.8 ± 0.3	52.0 ± 1.0	33.9 ± 2.4
T580	1805.0 ± 2.0	1301.0 ± 4.0	13.5 ± 0.0	47.0 ± 1.0	27.3 ± 0.7

## Data Availability

The data presented in this study are available on request from the corresponding author due to confidentiality.

## References

[1] Yu X.F., Shen X.Y., Wang S.S., Su Y., Zhao W.Z., Wei Y.H. (2022). Effect of Quenching and Tempering Treatment on Microstructure and Mechanical Properties of CSS-42L Bearing Steel. J. Mater. Eng. Perform..

[2] Chen X.F., Zheng L.J., Zhu Q.Y., Zhao S.T., Yu F., Liu M.H., Liu H.H., Zhang H., Xu H.B. (2022). Tempering influence on microstructural evolution and mechanical properties in a core of CSS-42L bearing steel. Mater. Sci. Eng. A.

[3] Zheng K., Cao W.Q., Yu F., Wang C.Y., Zhong Z.Q., Xu H.F. (2022). The research status and progress of high temperature stainless carburized bearing steel. Iron Steel.

[4] Bhadeshia H. (2012). Steels for bearings. Prog. Mater. Sci..

[5] Guan J., Wang L., Zhang Z., Shi X., Ma X. (2018). Fatigue crack nucleation and propagation at clustered metallic carbides in M50 bearing steel. Tribol. Int..

[6] Nygaard J.R., Rawson M., Danson P., Bhadeshia H. (2014). Bearing steel microstructures after aircraft gas turbine engine service. Mater. Sci. Technol..

[7] Trivedi H.K., Forster N.H., Rosado L. (2011). Rolling contact fatigue evaluation of advanced bearing steels with and without the oil anti-wear additive tricresyl phosphate. Tribol. Lett..

[8] Wang F.F., Li Q.S., Zheng L.J., Zhang F.X., Zhang H. (2018). Microstructure and corrosion characterization of Cr film on carburized CSS-42L aerospace bearing steel by filtered cathodic vacuum arc deposition. Coatings.

[9] Wang F.F., Zhang F.X., Zheng L.J., Zhang H. (2017). Structure and corrosion properties of Cr coating deposited on aerospace bearing steel. Appl. Surf. Sci..

[10] Forster N.H., Rosado L., Ogden W.P., Trivedi H.K. (2010). Rolling Contact Fatigue Life and Spall Propagation Characteristics of AISI M50, M50NiL, and AISI 52100, Part III: Metallurgical Examination. Tribol. Trans..

[11] Chen H., Zeng T.Y., Shi Q.Q., Wang N.M., Zhang S.Z., Yang K., Yan W., Wang W. (2023). Microstructure evolution and mechanical properties during long-term tempering of a low carbon martensitic stainless bearing steel. J. Mater. Res. Technol..

[12] Maloney J.L., Tomasello C.M. (1995). Case Carburized Stainless Steel Alloy for High Temperature Applications. U.S. Patent.

[13] Tomasello C.M., Burrier H.I., Knepper R.A., Balliett S., Maloney J.L., Beswick J.M. (2002). Progress in the evaluation of CSS-42L^(TM)^: A high performance bearing alloy. Bearing Steel Technology.

[14] Burrier H.I., Milam L., Tomasello C.M., Balliett S.A., Maloney J.L., Ogden W.P. (1998). Development of CSS-42L^(TM)^, a high performance carburizing stainless steel for high temperature aerospace applications. Proceedings of the Symposium on Bearing Steels: Into the 21st Century ASTM Committee A-1 on Steel.

[15] Uehara T., Watanabe R., Nakama N. (1994). Method of Manufacturing High Strength and High Toughness Stainless Steel.

[16] Wang W., Yang M.X., Wang J., Xie J.J., Wang J., Zhang Z.H., Zhou L.L., Wu X.L., Cao W.Q., Yuan F.P. (2024). Ultrahigh surface hardness and excellent hardness gradient induced by ultrafine dual-carbides in a novel stainless bearing steel. J. Mater. Res. Technol..

[17] Zheng K. (2025). Research on Strengthening-Toughening and Anti-Fatigue Performance of 17Cr14Co13Mo5Ni2W1V1Nb High-Temperature Bearing Steel.

[18] Xiao M.G., Lü X.Y., Li D.h., Li S.h., Zhao K.Y., Yang M.S. (2019). Carbides precipitation and their evolution of Cr15Co10Mo5-alloyed heat-resistant bearing steel after tempering at different temperatures. J. Iron Steel Res. Int..

[19] Zheng K., Zhong Z.Q., Wang H., Xu H.F., Yu F., Wang C.Y., Wu G.L., Liang J.X., Godfrey A., Cao W.Q. (2023). Obtaining excellent mechanical properties in an ultrahigh-strength stainless bearing steel via solution treatment. Metals.

[20] Niu G., Tang Q., Zurob H.S., Wu H., Xu L., Gong N. (2019). Strong and ductile steel via high dislocation density and heterogeneous nano/ultrafine grains. Mater. Sci. Eng. A.

[21] Dini G., Ueji R., Najafizadeh A., Monir-Vaghefi S.M. (2010). Flow stress analysis of TWIP steel via the XRD measurement of dislocation density. Mater. Sci. Eng. A.

[22] (2021). Metallic Materials—Tensile Testing—Part 1: Method of Test at Room Temperature.

[23] Zhang Y.P., Zhan D.P., Qi X.W., Jiang Z.H., Zhang H.S. (2019). The role of twins during the aging process of secondary hardening ultrahigh-strength steel. Mater. Lett..

[24] Lü X.Y., Wu Z.W., He X., Li J., Li S.H., Yang M.S., Zhao K.Y. (2020). Effect of deep cryogenic treatment on martensitic lath refinement and nano-twins formation of low carbon bearing steel. J. Iron Steel Res. Int..

[25] Zhang Y.P., Zhan D.P., Qi X.W., Jiang Z.H. (2018). Austenite and precipitation in secondary-hardening ultra-high-strength stainless steel. Mater. Charact..

[26] Zhang Y.P., Zhan D.P., Qi X.W., Jiang Z.H. (2019). Effect of tempering temperature on the microstructure and properties of ultrahighstrength stainless steel. J. Mater. Sci. Technol..

[27] Liu T., Cao Z., Wang H., Wu G., Jin J., Cao W. (2020). A new 2.4GPa extra-high strength steel with good ductility and high toughness designed by synergistic strengthening of nano-particles and high-density dislocations. Scr. Mater..

[28] Okayasu M., Motojima J. (2010). Microstructure-dependent hydrogen diffusion and trapping in high-tensile steel. Mater. Sci. Eng. A.

[29] Thomas G. (1978). Retained austenite and tempered martensite embrittlement. Metall. Trans. A.

[30] Zhang B.D., Li C.T., Fang K.W., Luo K.J., Wang L., Wu H.C., Fei F. (2021). Effect of tempering temperature on mechanical properties and stress corrosion sensitivity of 42CrMo4 high-strength steel. Mater. Rep..

